# The availability and distribution of health services and resources across different regions in Afghanistan

**DOI:** 10.3389/fpubh.2024.1371104

**Published:** 2025-01-24

**Authors:** Nelly Hegazy, Sherif El Deeb, Marwa Rashad Salem, Jerome A. Shaguy, Ramesh Nassery Mohammed, Abdullah Khawari, Jamshed Tanoli, Alaa Abouzeid

**Affiliations:** ^1^Public Health and Community Medicine, Faculty of Medicine, Helwan University, Cairo, Egypt; ^2^Community Medicine Research Department, National Research Centre, Giza, Egypt; ^3^Public Health and Community Medicine, Faculty of Medicine, Cairo University, Cairo, Egypt; ^4^The World Health Organization, Kabul, Afghanistan

**Keywords:** HeRAMS, Afghanistan, health services, availability, functionality

## Abstract

**Background:**

Ensuring equitable access to healthcare services is fundamental to a robust healthcare system, especially during humanitarian crises. This study analyzes the availability and distribution of health resources across Afghanistan, aiming to provide a data-driven understanding of healthcare resource access and identify potential disparities, a critical aspect of effective humanitarian response planning.

**Methods:**

Principal investigators collated related data and literature from databases and data warehouses in a systematic approach using search strings and collection tools to query databases and available data warehouses to assess the availability and distribution of health services and resources across different regions in Afghanistan, with the principal database queried being the Afghanistan Health Resources Availability database (HeRAMS), an electronic and web-based system conceived by the World Health Organization. An Excel version was sourced.

**Results:**

The sub-health center represents 31.1% of the health facilities, followed by the basic health center (22.5%) and the mobile health team (17.1%). More than 85% of these facilities are fully operational, with the highest percentage observed in the Southern and Northeastern regions at 96.8%, followed closely by the central highland and Southeastern regions. Outpatient services for primary care are notably prevalent in the Northeastern, Southeastern, Southern, Eastern, and Northern region, conversely, the Capital and Central Highland regions demonstrate the lowest provision of primary care services. Antenatal care services are accessible at a level exceeding 70% in nearly all regions, with the highest accessibility in the Northeastern region at 91.3%, the prevalence of non-communicable illness clinics was observed to be below 50%, with the highest availability in the Southern region at 49.4%, followed by the Southeastern region. In terms of sanitation facilities, availability surpasses 70% across Afghanistan, with the highest observed in the Northeastern region at 89.2%.

**Conclusion:**

The study highlights significant disparities in healthcare access across various regions, with notable challenges in the availability of critical services. Furthermore, the study underscores the significant impact of financial constraints and equipment shortages on the functionality of healthcare facilities, particularly in the Northeastern and Western regions. This analysis emphasises the need for targeted resource allocation and infrastructure improvements to address inequities in access to essential healthcare services, particularly for underserved populations, thereby facilitating the achievement of equitable health outcomes in Afghanistan.

## 1 Introduction

Access to healthcare services and the equitable distribution of healthcare resources are critical foundations of a well-functioning healthcare system (1). Ensuring that healthcare services are accessible to all individuals, regardless of their location, is vital for promoting public health and achieving optimal health outcomes, which aligns with the global health initiative of “Health for All,” which advocates for universal access to essential healthcare services as a fundamental human right (2, 3). The healthcare system in Afghanistan has struggled to be sustainable, facing persistent issues of inadequate funding, limited resources, a shortage of qualified staff, insufficient equipment, and a lack of essential medical supplies (4). Between 2021 and 2022, the situation worsened significantly, with the healthcare system barely managing to function due to a severe lack of funding (5). In the long term, increased allocation of domestic resources to health services is key for ensuring the sustainability of the country’s health system (6).

Recent research conducted by Médecins Sans Frontières (MSF), an international medical humanitarian organization, reveals that Afghans continue to face challenges in accessing healthcare. This is due to a combination of increasing poverty and a further weakening of the public health system, which exacerbates the existing health needs (7). An alarming 88% of survey participants reported delaying, suspending, or forgoing medical care due to various barriers, representing a 14.3% increase from 2021. This suggests that while there may be greater freedom of movement in 2022, seeking healthcare remains a challenge for many. Shockingly, 52% of these respondents believe that their relatives passed away due to a lack of or delayed access to healthcare in the past year (8).

In the context of humanitarian response, planners and policymakers typically require real-time analyses of resources in three key dimensions of public health management: what, when, and where. This study aims to assess the availability and distribution of health services and resources across different regions in Afghanistan by employing the Health Resources Availability Mapping System (HeRAMS) (9), a World Health Organization-developed tool that utilizes standardized metrics to assess medical and health resource availability in emergency contexts. HeRAMS, currently deployed in 21 countries, is being used in Afghanistan, where 227 contributors update data on over 4,760 health facilities, providing valuable insights into the country’s health resource landscape. (REFERENCE). It also seeks to identify the primary obstacles that hinder the full functionality of these services. Furthermore, the study aims to highlight the regions most affected by shortages in health resources and services, providing valuable guidance to decision-makers in resource allocation. By shedding light on these critical aspects of healthcare provision, this study strives to offer valuable insights to ongoing efforts aimed at improving healthcare delivery and reducing disparities in access to care.

## 2 Methods

The study utilized a filtered and systematic review approach that largely focused on querying the HeRAMS database through searches that investigated the following questions: what resources are available, when are they available and where are they available? These questions allowed investigators the leverage to examine supply-side issues with regard health resource availability. Data defined by the dimensions mentioned above was dumped into an excel spreadsheet. Following this sourcing, the first step was to display the data in discernable frameworks for analysis. Investigators used excel functions to disaggregate and filter the database for themes of issues and patterns of information. This was an iterative process, involving several rounds of review. Each iteration filtering the supply side issue in more focused dimensions which became themes. The first iteration disaggregated levels of functionality as a measure of general resource availability status. Once functionality was established, the location dimension was examined. Which region and or province has the most severe resource gaps. An itemization of the actual gaps in human resource and physical infrastructure gaps terms.

These layers of queries and analysis provided the descriptions of the availability issues, issues that are displayed and described in the results section below.

### 2.1 Results

**Figure 1:**
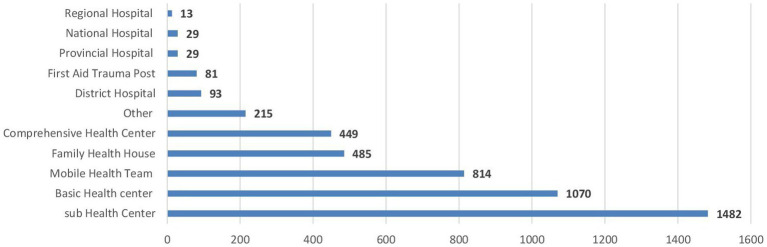
The bar chart represents the health facility types across Afghanistan.

Since November 2021, the Health Resources and Services Availability Monitoring System (HeRAMS) has been in place in Afghanistan, enabling the evaluation of 4,760 health facilities nationwide. The sub-health center represents 31.1% (1,482 centers) of the health facilities, followed by the basic health center 22.5% (1,070 centers) and the mobile health team 17.1% (485 centers), as shown in Figure 1. 42,691 health staff 58.5% males and 41.5% females, are providing medical services across the country. Male and female health staff include general practitioners, specialists 80.1% (1,123) male and 19.9% (279) female, nurses 66.95% (4,770) male and 33.05% (2,355) female, and other staff 62.35% (14,089) male and 37.65% (8,507) female. However, nearly all midwives are female 99.41% (5,000), and 91.71% (2,091) of technicians and pharmacists are male *(nontabulated)*.

Fixed health facility structure constitutes 53.71% (2,553 health facilities); temporary structures, like tents, are 18.56% (882 health facilities); mobile clinics represent 16.24% (772 health facilities); and 11.49% (546 health facilities) are parts of other facility buildings. 90.3% (3,947 health facilities) have full availability of health records, whereas partial availability is in 5.7% (255 health facilities). Health Emergency Response Project (HER), funded by the World Bank, supports more than half of the health facilities in Afghanistan *(nontabulated)*.

**Figure 2:**
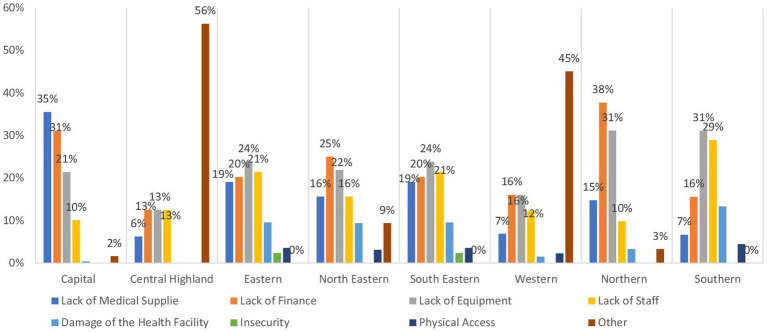
Bar chart demonstrating the main causes of dysfunctionality by region in Afghanistan.

Table 1 provides an overview of the operational status of health facilities across regions in Afghanistan, indicating a significant variation in their functionality. It is evident that more than 85% of these facilities are fully operational, with the highest percentage observed in the Southern and Northeastern regions at 96.8%, followed closely by the central highland and Southeastern regions at 95.5%. In contrast, approximately 14.5% of health facilities in the Capital region are reported to be partially functioning. Conversely, around 24.8 and 23.2% of health facilities in the Northeastern and Western regions, respectively, are reported to be partially damaged, followed by the Northern region at 20.4%. As depicted in Figure 2, the primary obstacles hindering the operational efficacy of health facilities in the Northeastern (mainly in Badakhshan, and Baghlan provinces) and Northern (especially in Balkh province) regions predominantly stem from insufficient finance and inadequate equipment, collectively accounting for 50% or more. Conversely, in the Western region (mainly in Ghor Badghis and Farah provinces).

**Table 1:** Percent distribution of health facilities by health facilities status across regions in Afghanistan (*n* = 4,760).

Regions	Status of Health facilities													
	Functionality						Building Damage							
	Fully Functioning		Partially functioning		Not Functioning		Insignificant damage		Partially damaged		Major or fully damaged		Mobile clinics (Not relevant)	
	No	%	No	%	No	%	No	%	No	%	No	%	No	%
Capital	596	85.5	101	14.4	0	0	573	82.2	75	10.7	0	0	49	7
Central Highland	243	95.6	3	1.1	8	3.1	197	77.2	38	14.9	0	0	20	7.8
Eastern	558	94.4	33	5.5	0	0	464	78.5	35	5.9	0	0	92	15.5
Northeastern	470	96.7	14	2.8	2	0.41	329	67.4	121	24.8	1	0.2	37	7.5
Southeastern	470	95.5	22	4.4	0	0	397	80.3	32	6.4	2	0.4	63	12.7
Western	540	86.8	78	12.5	4	0.6	403	64.6	145	23.2	1	0.2	74	11.8
Northern	552	95.6	21	3.6	4	0.7	389	67.3	118	20.4	1	0.2	70	12.1
Southern	651	96.8	21	3.1	0	0	557	82.7	40	5.9	1	0.2	75	11.1

**Figure 3:**
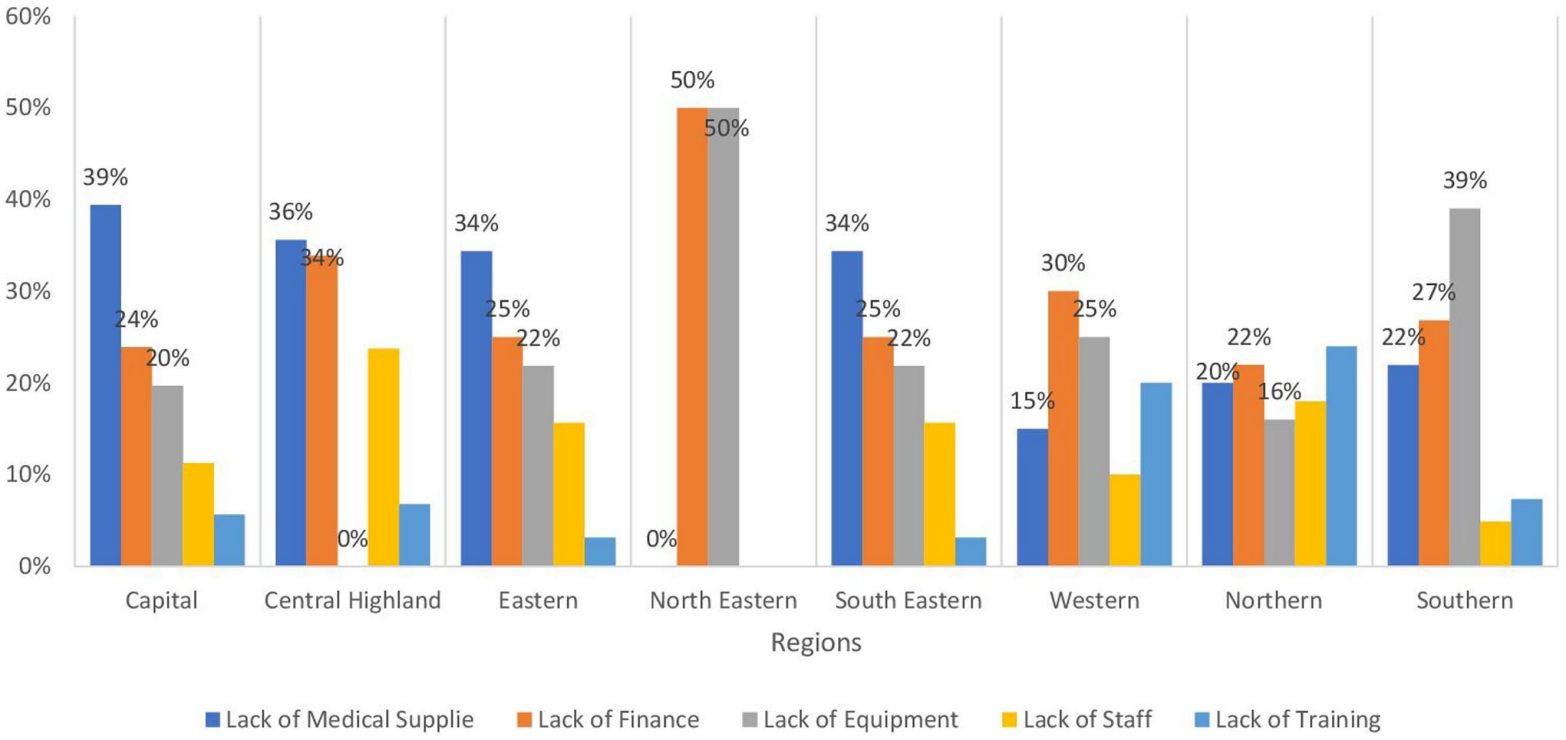
Bar chart demonstrating the barriers to outpatient services for primary by regions in Afghanistan.

Table 2 provides insights into the availability of essential general clinical services across different regions of Afghanistan, which is crucial for addressing the diverse health needs of the population, from primary care to specialized medical treatment. Outpatient services for primary care are notably prevalent in the Northeastern, Southeastern, Southern, Eastern, and Northeastern regions, with percentages of 89.6, 86.1, 84.6, 82, and 78.5%, respectively. Conversely, the Capital and Central Highland regions demonstrate the lowest provision of primary care services, each at 64%. More than 35% of this deficiency in both regions can be attributed to inadequate medical supplies, followed by financial inadequacies, as illustrated in Figure 3. Additionally, outpatient department services for primary care are accessible at rates exceeding 70% in the southeastern, southern, eastern, and northeastern regions at 77.9, 72.3, 70.2, and 70.1% respectively, whereas the Central Highland region reports a markedly lower percentage at 18.3%. Recognition of danger signs is prevalent at rates exceeding 70% in the Central Highland and Northeastern regions, with percentages of 78.1 and 73.7% respectively, while the Western and Capital regions exhibit the lowest availability at 54.2 and 56.9%, respectively. Referral capacity is highest in the Northeastern region at 63.2%, and in the Central Highland region at 57.8% and lowest in the Northeastern and Capital regions at 28.6 and 38.3%, respectively.

**Table 2:** Percent distribution of general clinical services availability across regions in Afghanistan (*n* = 4,760).

Region	Percent of the availability of General clinical services									
	Outpatient services for primary care	Outpatient department for 2ry care	Home visits	Intensive care units	Blood bank services	Inpatient critical care management	Referral capacity	WHO Basic emergency care	Recognition of Danger signs	Procedures for mass casualty scenarios
	%	%	%	%	%	%	%	%	%	%
Capital	64.6	53.8	20.9	35.6	5.7	9.6	38.3	38.1	56.9	19.2
Central Highland	64.3	18.3	46.5	29.1	0.81	4.3	57.8	20.6	78.1	5.8
Eastern	82.0	70.2	12.1	8.8	2.5	18.8	50.5	49.1	66.8	19.9
Northeastern	89.6	70.1	50.8	31.5	5.5	16.3	63.2	33.4	73.7	8.5
Southeastern	86.1	77.9	30.5	44.4	3.6	8.5	53.3	51.9	65.5	19.7
Western	66.3	67.4	39	21.3	2.9	9.6	47.5	41.1	54.2	26
Northern	78.5	62.9	32.3	38.4	3.5	10.5	28.6	40.7	68.5	12.1
Southern	84.6	72.3	40.7	35.2	1.6	17.7	54.02	35.5	60.7	16.8

The provision of intensive care units is notably higher in the Southeastern region at 44.5% and the Northeastern region at 38.4%, whereas the Eastern region reports the lowest availability at 8.8%. WHO Basic emergency care is predominantly available in the Southeastern region at 51.9% and the Eastern region at 49.07%, but only at 20.6% in the Central Highland region.

Other clinical services display considerably lower availability, with percentages below 50% across regions. Home visits are conducted in the Northeastern, Central Highland, and Southern regions at rates of 50.8, 46.5, and 40.7% respectively, whereas the Western and Capital regions report lower percentages at 12 and 20.9%, respectively. The delivery of inpatient critical care management is notably below 20% across regions, with the highest availability in the Eastern region at 18.8%, followed by the Southern and Northeastern regions at 17.7 and 16.3%, respectively.

Procedures for mass casualty scenarios are executed in only 26% of the health facilities in the Western region and at nearly equal percentages of 19% in the Eastern, Southeastern, and Capital regions, while only 5% of health facilities in the Central Highland region provide these procedures. Regarding blood bank services, their availability is notably scarce, with the highest in the capital region at 5.7% and the Northeastern region at 5.5%, while the Central Highland region reports a significantly lower percentage at 0.2%.

**Figure 4:**
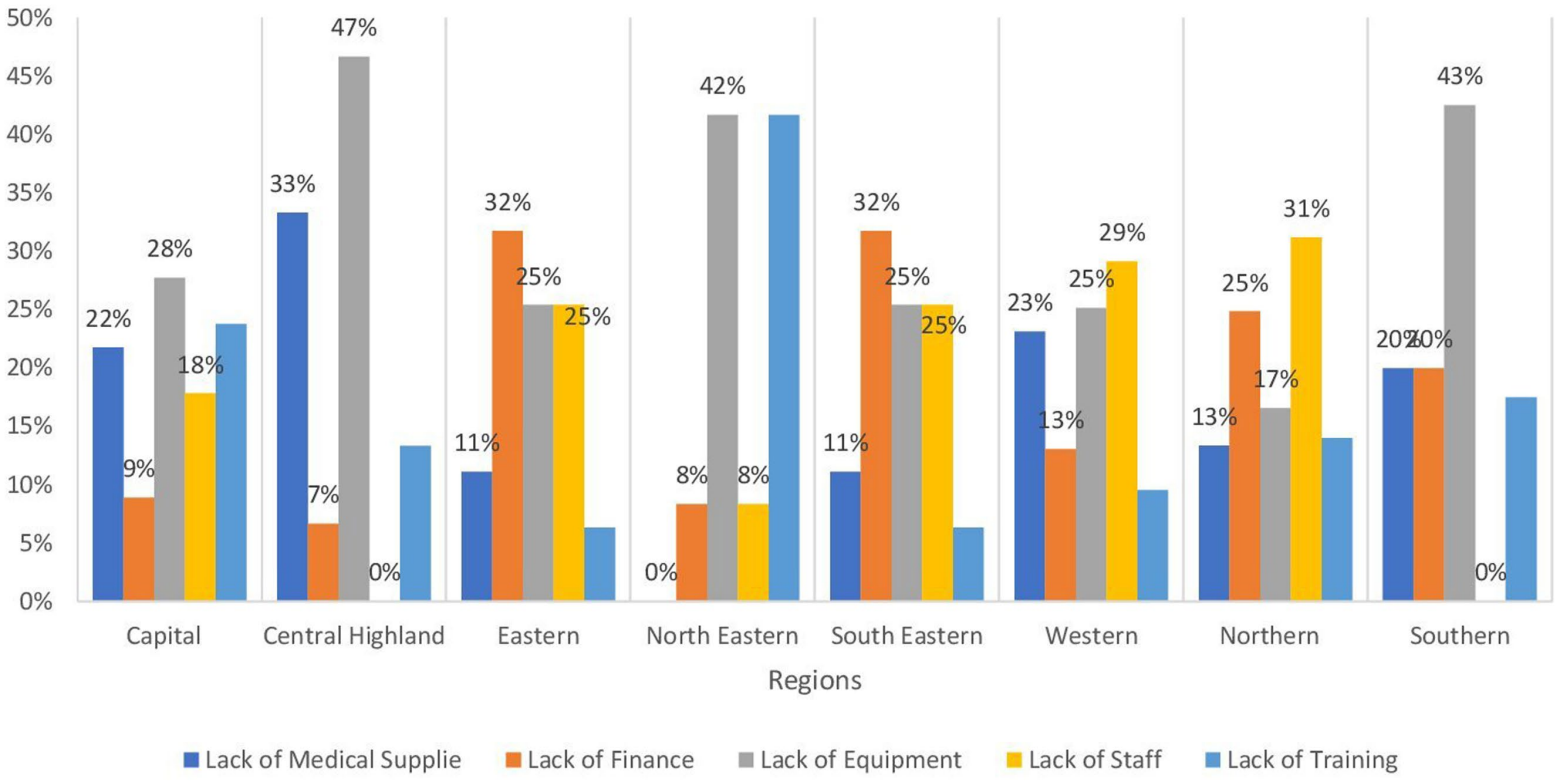
Bar chart demonstrating the main barriers expanded programs on Immunization by regions in Afghanistan.

**Figure 5:**
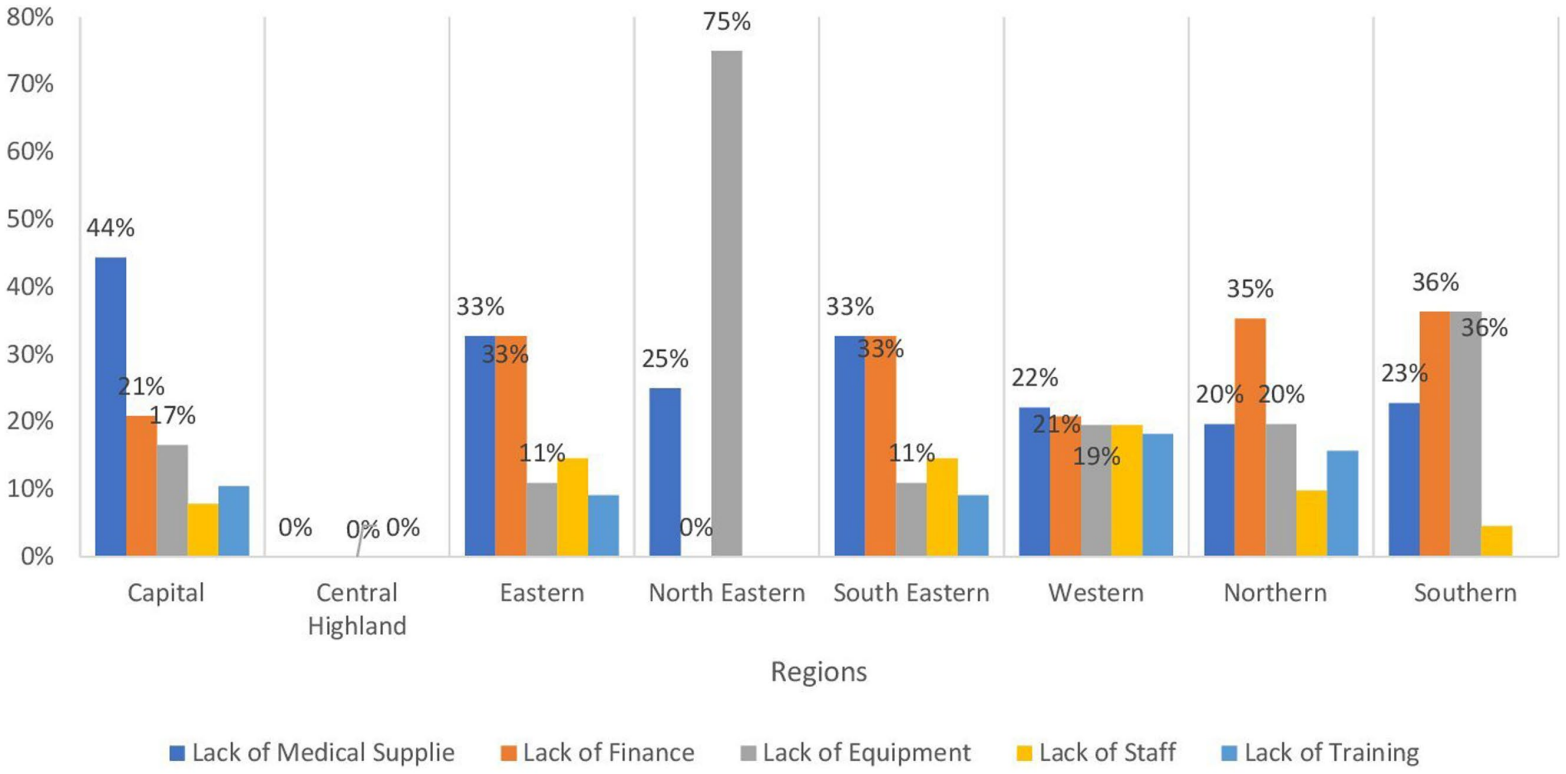
Bar chart demonstrating the main barriers to Integrated Management of Newborn and Childhood illness under 5 clinics by regions in Afghanistan.

Table 3 presents the availability of vital health services for children across different regions in Afghanistan. Ensuring that children have access to these services is paramount for promoting their health and well-being and reducing the high rates of child mortality and morbidity that persist in the country. In terms of the Expanded Program of Immunization (EPI), it is notably accessible in over 70% of health facilities in the Southern, Northeastern, and Southeastern regions at 78.7, 73.5, and 73.1%, respectively. However, the availability is lower in the Western region at 46.2%, where 29% is due to the lack of staff, then lack of equipment and medical supplies at 25 and 23% respectively, whereas in the Northern region at 48.08% and is attributed to lack of staff 31% followed by 25% lack of Finance, as shown in Figure 4. Similar trends are observed in community mobilization for EPI. The Integrated Management of Newborn and Childhood Illness (IMNCI) is widely available in almost all regions at a level exceeding 70%, with the highest provision in the Central Highland region followed by the Southeastern and Southern regions at 85%. In contrast, the Capital and Eastern regions exhibit the most limited availability of IMNCI, with rates of 67.1 and 69.04%, respectively. In the Capital region, a predominant factor contributing to the low availability of IMNCI is the inadequacy of medical supplies, accounting for 64% of the barriers. Meanwhile, in the Eastern region, 45% of the constraints affecting IMNCI availability are attributed to a deficiency in training, as evidenced in Figure 5. The management of severe childhood diseases is offered in 50% of health facilities in the Southern region, with lower availability across the rest of the regions. The Northern region demonstrates the lowest availability at 22.4%, followed by the Central Highland region at 24.08%.

**Table 3:** Percent distribution of the availability of Health services provided to children across regions in Afghanistan (*n* = 4,760).

Region	Percent of the availability of health services									
	EPI	Community mobilization on EPI	IMNCI^*^ under 5 clinic	Management of severe diseases	Growth monitoring at PHC	Screening acute malnutrition at the community level	OPD for MAM^**^	OPD for SAM^***^	IDP For SAM^****^	IEC & IYCF^*****^ for children
	%	%	%	%	%	%	%	%	%	%
Capital	58.7	52.7	67.1	38.41	76.7	65.2	66.9	63.2	18.8	70.5
Central Highland	59.5	72.8	91.09	23.08	89.07	90.2	50.6	85.4	15.5	87.8
Eastern	67.6	67.1	69.04	49.1	80.7	71.9	68.1	75.1	10.5	66.1
North Eastern	73.5	68.1	75.4	32.5	86.3	86.7	77.8	79.1	11.4	90.08
South Eastern	73.1	72.1	85.9	44.9	87.7	85.5	75.1	77.8	12.5	83.3
Western	46.2	47.9	73.9	32.2	77.9	74.2	55.5	58.5	7.7	76.2
Northern	48.08	49.4	73.7	22.4	88.1	76.4	79.3	74.6	20.9	73.2
Southern	78.7	79.3	85.6	50.9	87.3	79.7	83.4	85.5	12.6	85.7

* IMNC, integrated management of Newborn and Childhood illness.

** OPD for MAM, outpatient department for moderate malnutrition.

*** OPD for SAM, outpatient department for severe malnutrition.

**** IDP For SAM, inpatient department for severe malnutrition.

***** IEC & IYCF, information, education & communication on infant and young child feeding.

In terms of child growth and development, growth monitoring at primary healthcare facilities and screening for acute malnutrition at the community level are widely available in all regions at levels exceeding 70%. The Central Highland region demonstrates the highest availability at 89.7 and 90.2% respectively, while the Capital region shows the lowest availability for both services, at 76.6 and 65.2%, respectively. Information, education, and communication on infant and young child feeding exhibit good availability in nearly all regions, with the highest in the Northeastern region at 90%, except for the Eastern region, which demonstrates 66% availability.

Outpatient departments for moderate malnutrition are available at levels exceeding 70% in the Southern, Northern, Northeastern, and Southeastern regions, with the Central Highland region joining the top regions in demonstrating the highest availability. Conversely, the Central Highland and Western regions show the lowest availability of services for moderate malnutrition at 50.6 and 55.5%, respectively. For severe malnutrition, the Western region demonstrates a 58.5% availability, and the Capital region shows a 63.2% availability. In contrast, the availability of inpatient departments for severe malnutrition is notably lower, below 20% across all regions, with the Northern region exhibiting the highest availability at 20.9% and the Western region demonstrating the lowest at 7.7%.

**Figure 6:**
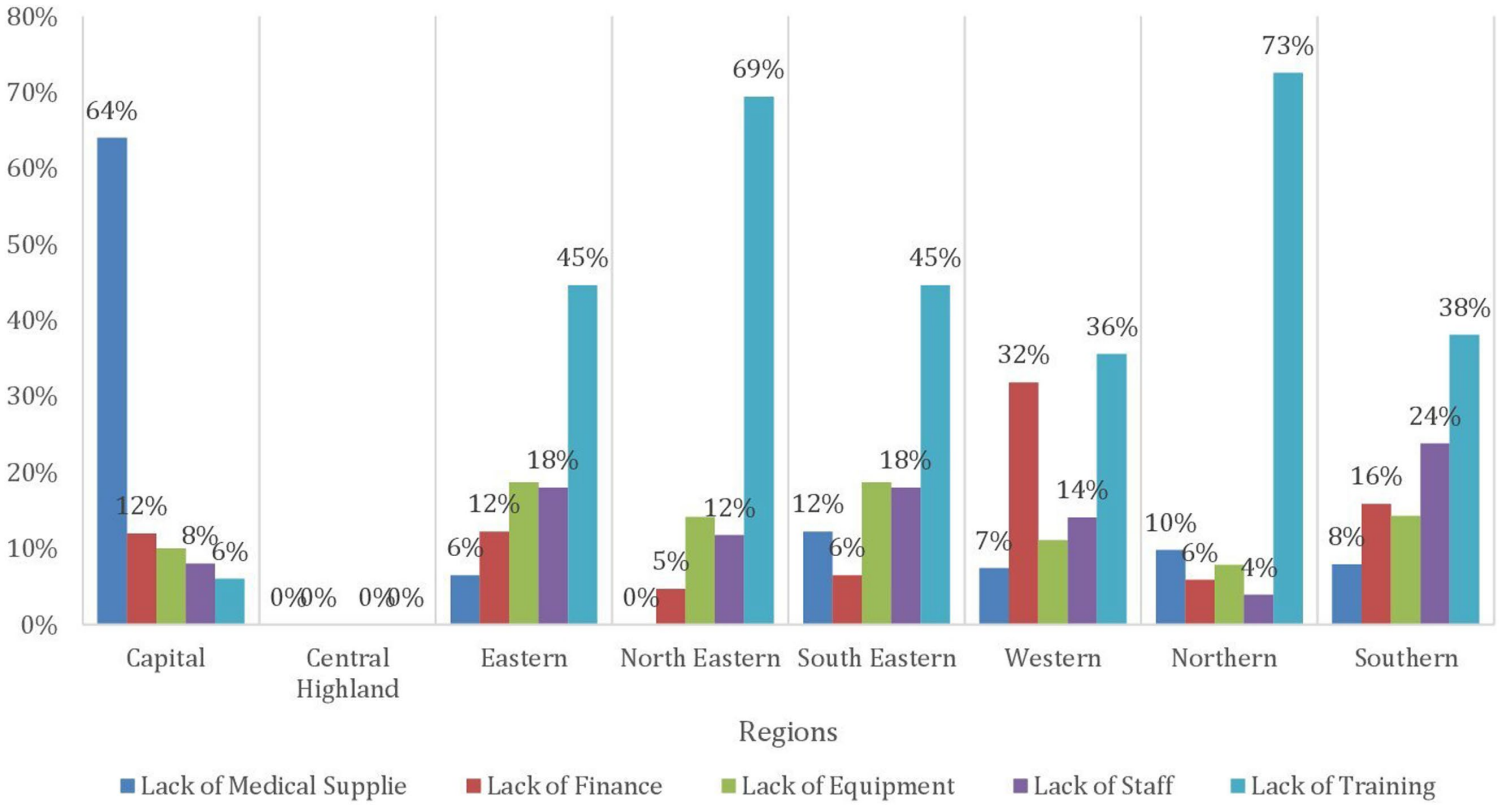
Bar chart demonstrating the main barriers to antenatal care by regions in Afghanistan.

Table 4 details the accessibility of maternal and newborn health services across various regions, which is crucial for reducing maternal and neonatal mortality rates, which remain unacceptably high in the country. Antenatal care services are accessible at a level exceeding 70% in nearly all regions, with the highest accessibility in the Northeastern region at 91.3% and the Central Highlands region at 90.6%, while the Capital Region exhibits the lowest availability at 71.6%, where approximately 44% of the obstacles to availability are attributed to the scarcity of medical supplies, as shown in Figure 6. Similarly, skilled birth attendance during childbirth demonstrates the highest accessibility in the Northeastern region at 90.2%; however, the Eastern and Capital regions display lower percentages at 66.6 and 68.6%, respectively. Clean home deliveries are most prevalent in the Central Highlands and the Southern Highlands, with accessibility at 87.04 and 85.2% respectively, while the Eastern (50.5%) and Capital regions (59.7%) reveal the lowest percentages.

**Table 4:** Percent distribution of the availability of maternal and newborn health services across regions in Afghanistan (*n* = 4,760).

Region	Percent of the availability of maternal and newborn health services						
	Antenatal care	Skilled Birth attendance during child birth	Clean home deliveries	Basic emergency obstetric care	Comprehensive emergency obstetric care	Postpartum care	Family planning
	%	%	%	%	%	%	%
Capital	71.6	68.6	59.7	51.3	35.9	71.08	72.6
Central Highland	90.6	86.6	87.04	88.2	19.3	89.8	91.09
Eastern	83.0	66.6	50.5	57.8	26.5	77.6	83.2
Northeastern	91.3	90.2	76.6	71.9	34.1	87.6	92.1
Southeastern	88.1	87.3	67.8	74.7	40.5	87.3	90.4
Western	86	77.07	77.7	61.6	24.8	84.3	90.4
Northern	83.8	80.9	70.7	78.6	23.2	82.8	86.1
Southern	88.1	83.9	85.2	73.8	20.6	86.4	88.3

**Figure 7:**
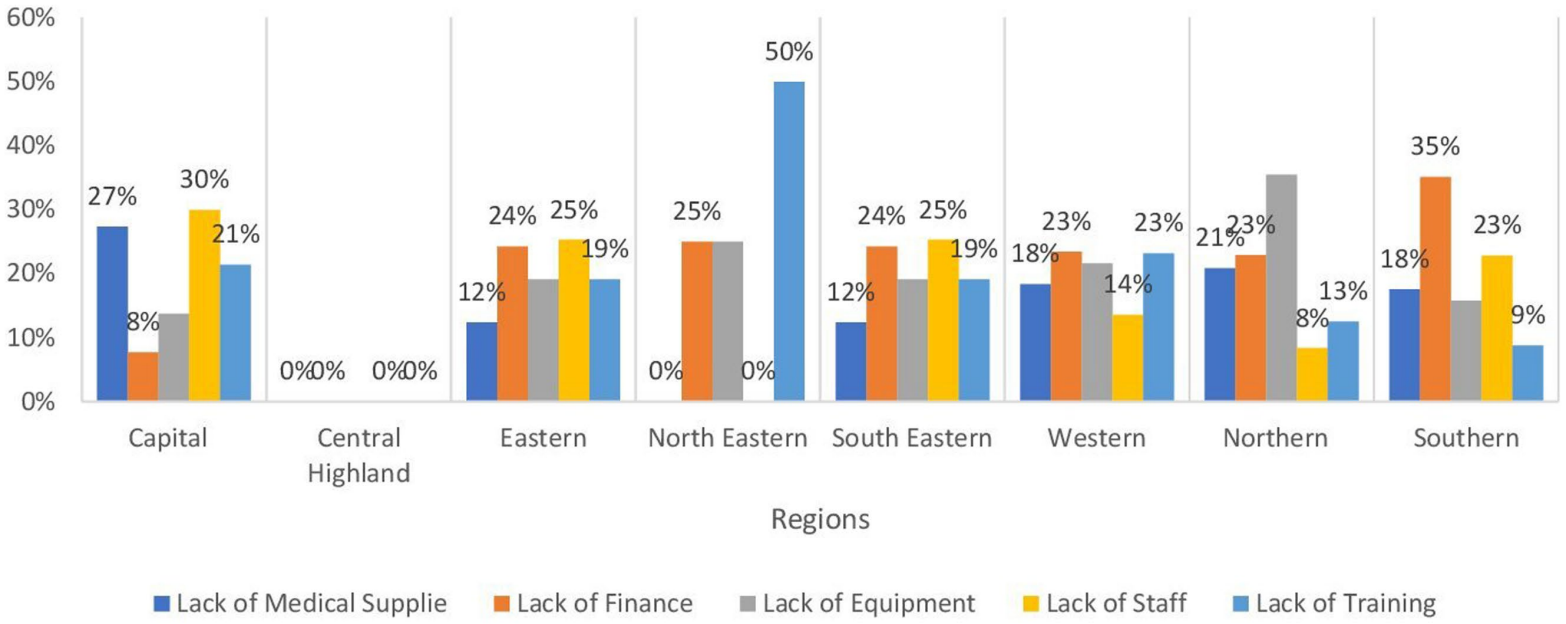
Bar chart demonstrating the main barriers to basic emergency obstetric care by regions in Afghanistan.

In terms of emergency obstetric care, basic services are available at a percentage above 70% in the Central Highland region (the highest at 88.2%), followed by the Northern, Southeastern, Southern, and Northeastern regions respectively, whereas the Capital (51.3%) and Eastern (57.8%) regions rank lower in availability. The principal obstacle to the limited availability of basic emergency obstetric care in the Capital and Eastern regions is the inadequacy of trained staff, accounting for 30 and 25%, respectively. This is closely followed by a deficit in medical supplies, which contributes to 27% of the challenge in the capital region, while financial insufficiency accounts for 24%, as delineated in Figure 7. Meanwhile, comprehensive emergency obstetric care is attainable at a substantially lower availability, below 50%, with the highest availability in the Southeastern region at 40.5%, followed by much lower availability across regions until the Central Highlands region offering only 19.3% and the Southern region 20.6%.

Regarding postpartum care and family planning, these services are widely accessible in most regions at a level exceeding 70%, with the highest percentages in the Central Highland and Northeastern regions, apart from the Capital region, which shows 71.08% accessibility. Postpartum care is available at 76.6% in the Eastern region, while family planning accessibility is at 72.6% in the capital region.

Table 5 explores the availability of health services for the management of communicable diseases across different regions in Afghanistan, which continue to pose a significant public health challenge in the country, requiring robust interventions for prevention, treatment, and control. The accessibility to information, education, and communication related to communicable diseases is notably higher in the southeastern and capital regions, at 78.1 and 72.9%, respectively. Meanwhile, other regions exhibit availability levels of less than 70%, with the eastern and northern regions being the least accessible at 56.4 and 57.1%, respectively.

**Table 5:** Percent distribution of the availability of health services for communicable diseases across regions in Afghanistan (*n* = 4,760).

Region	Percent of the availability of Health services for communicable diseases							
	Management of severe/complicated communicable diseases	^*^IEC on local priority diseases	Vector control	Syndromic surveillance	Isolation unit or room	Malaria at PHC	Tuberculosis at PHC	MDRTB^**^ at PHC
	%	%	%	%	%	%	%	%
Capital	38.4	63.6	23.85	21.2	26.8	60.7	27.4	12.7
Central Highland	23.1	78.1	48.99	42.5	9.6	78.9	36.4	28.7
Eastern	49.1	57.1	43.15	14.2	11.8	65.1	29.9	9.1
Northeastern	32.5	65.2	26.65	28.1	14.8	78.5	31.	14.6
Southeastern	44.9	72.9	27.29	27.7	14.3	78.6	30.9	33.1
Western	32.2	56.4	10.21	36.63	15.4	56.2	21.2	24.4
Northern	22.4	66.4	7.1	37.1	16.1	59.2	20.8	17.2
Southern	50.9	67.7	38.1	31.1	19.8	71.1	28.5	35.4

* IEC, information, education & communication.

** MDRTB, multidrug-resistant tuberculosis.

The availability of management for severe or complicated communicable diseases is generally lower than 50% across regions, with the highest availability in the Southern region at 50.9% and the Capital region at 49.1%, whereas the Northern region exhibits the lowest availability at 9.6%. Syndromic surveillance is applicable at a level lower than 50%, with the highest prevalence in the Northern region at 42.5% and the lowest in the Western region at 14.2%. Vector control services are present at a level of less than 50% across regions, with the Eastern region having the highest prevalence at 48.9% and the Northern region at the lowest with only 7.1%.

In terms of specific communicable diseases, the provision of services for Malaria at the primary healthcare level is prevalent at 78% in the Southeastern, Capital, and Eastern regions, with the northern region being the least accessible at 56.2%. Services for tuberculosis are less widely available, with the highest availability in the Northeastern region at 36.4%, followed by the Eastern region at 31%, and the lowest availability in the Northern and Western regions at 20.8 and 21.2%, respectively. Similarly, services for Multidrug-Resistant Tuberculosis are predominantly accessible in the Southern (35.4%) and Capital (33.1%) regions, with the Southeastern region being the lowest at 9.1%.

Table 6 examines the availability of health services for non-communicable diseases (NCDs), as the increasing burden of NCDs in the country necessitates expanded access to appropriate prevention, management, and treatment services. The prevalence of non-communicable illness clinics was observed to be below 50%, with the highest availability in the Southern region at 49.4%, followed by the Southeastern region at 48.1%, and the Northeastern region at 46.4%. In contrast, the Northern and Western regions demonstrate the lowest availability at 22%.

**Table 6:** Percent distribution of the availability of health services for noncommunicable diseases across regions in Afghanistan (*n* = 4,760).

Region	Percent of the availability of health services									
	NCD^*^ clinic	Hypertension	Asthma &COPD^**^	Diabetes	Physiotherapy services	Prosthetics & orthotics	Inpatient acute rehabilitation	Management of MD^***^	Inpatient care for MD	Psychological 1^st^ aid
	%	%	%	%	%	%	%	%	%	%
Capital	35.9	69.3	54.3	28.1	7.1	4.4	17.9	26.6	9.6	36.2
Central Highland	33.2	76.1	42.5	27.1	2.8	0.8	8.6	21.8	4.3	31.5
Eastern	45.8	83.2	49.9	11	3.5	3.8	19.9	51.1	18.8	35.1
Northeastern	46.4	91.7	65.7	21.6	4.5	3.9	13.7	37.1	16.3	28.1
Southeastern	48.1	86.9	61.7	21.3	10.1	3.2	20.3	52.1	8.5	8.5
Western	22.8	63.9	42.5	11.7	2.9	1.6	7.7	38.9	9.6	37.1
Northern	22.4	83	49.9	14.5	5.1	5.7	16.1	21.3	10.5	32.4
Southern	49.4	74.1	49.8	14.2	4.3	14.2	18.1	38.1	17.7	47.6

* NCD, noncommunicable diseases.

** COPD, chronic obstructive pulmonary diseases.

*** MD, mental disorders.

Regarding the provision of services for specific noncommunicable diseases, services for hypertension are widely available across regions, exceeding a 70% availability rate. The Northeastern region shows the highest applicability at 91.7%, while the capital region displays a lower rate of 63.9%. Conversely, accessibility to asthma services is highest in the Northeastern region at 65.7%, followed by the Southeastern region at 61.7%. Equally, the Western and Central Highland regions exhibit a 42.5% availability rate for these services. The availability of diabetes services, however, is notably lower, standing at less than 30%, with the capital and Central Highlands regions demonstrating the highest percentages at 28 and 27%, respectively.

Physiotherapy services are limited across the regions, with the Southeastern region having the highest availability at 10%. Similarly, the availability of prosthetics and orthotics is at its highest in the southern region, reaching 14%. Inpatient services for acute rehabilitation are most prevalent in the Southeastern and Eastern regions, with availability levels reaching nearly 20%.

**Figure 8:**
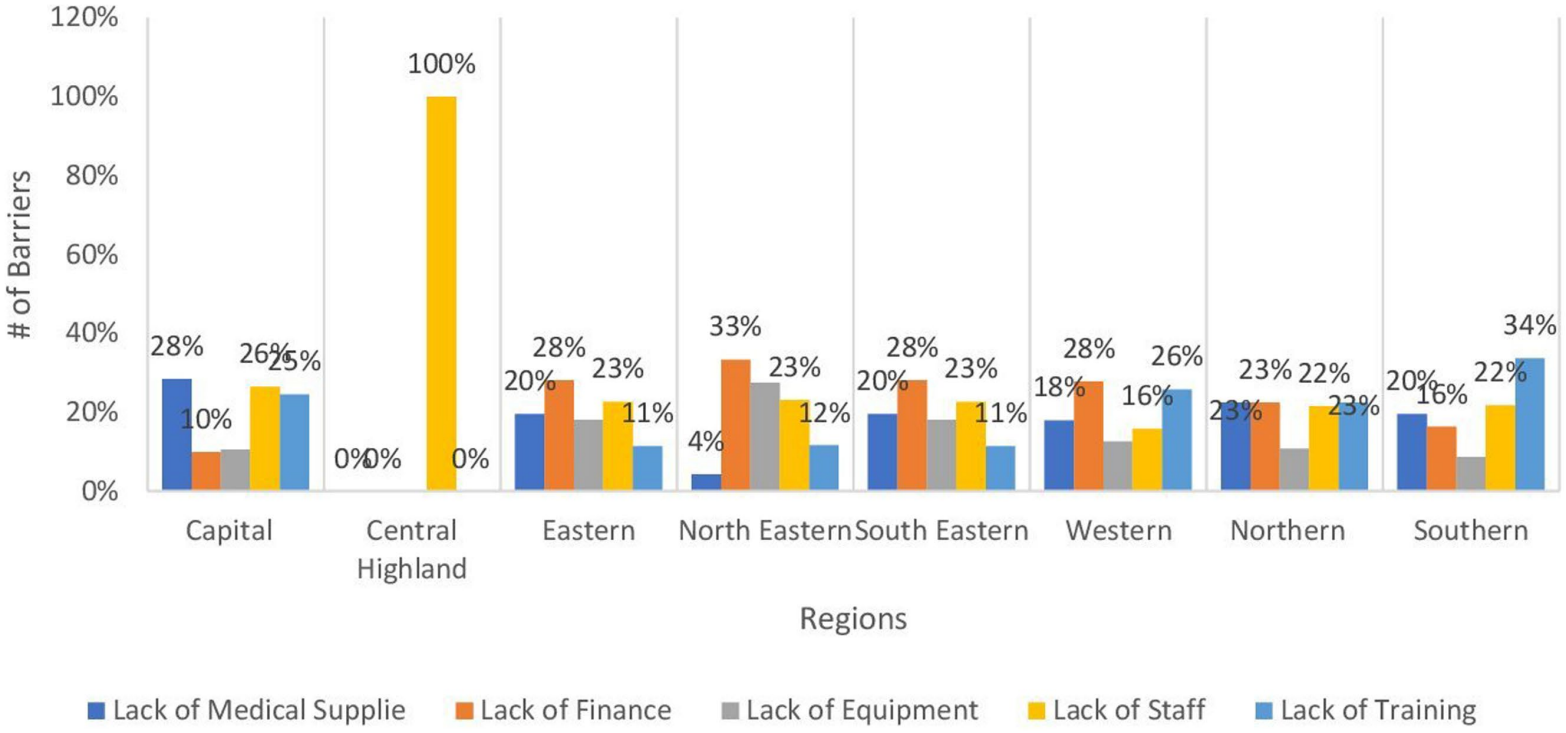
Bar chart demonstrating the main barriers to the management of mental disorders by regions.

In terms of mental health services, the availability of services across regions is consistently below 50%, with the Southeastern and Eastern regions demonstrating the highest availability at nearly 52%, while the Central Highland and Northern regions exhibit the least availability at 21%. The Central Highland region is predominantly hindered by the absence of staff, accounting for 100% of the barriers to availability. In contrast, the Northern regions exhibit similar percentages of approximately 23% for lack of medical supplies, finance, staff, and training, all representing primary obstacles to availability, as illustrated in Figure 8. Similarly, the accessibility of psychological first aid is highest in the southern region at 47.6%, followed by the western region at 37%, and lowest in the southeastern region at 8.5%. However, inpatient care for mental disorders lacks prevalence, with the Eastern, Southern, and Northeastern regions ranging from 16 to 18% availability, while the Central Highland region shows a 4.3% availability rate.

Table 7 highlights the availability and accessibility of sexual and reproductive health services across different regions in Afghanistan, which is vital for promoting women’s health, preventing sexually transmitted infections (STIs), and supporting victims of sexual violence. The availability of HIV counseling and testing services is observed to be below 50%, with the highest availability in the Southeastern region at 43% and the Northeastern region at 43%, while the Western, Northern, Capital, and Eastern regions demonstrate the lowest availability, ranging from 14 to 16%. Similarly, the availability of syndromic management for sexually transmitted infections displays varying availability, with the highest prevalence in the Central Highland region at 53.4% and the lowest in the Capital Region at 32.9%. In contrast, information, education, and communication on STIs and HIV are more widely available, with the Southeastern region leading at 81.8%, followed by the Northeastern region at 60.3%, and the Western region having the lowest availability at 41.9%.

**Table 7:** Percent distribution of the availability of health services for the management of sexual transmitted infections/HIV and sexual violence diseases across regions in Afghanistan (*n* = 4,760).

Region	Percent of the availability of health services			
	HIV counseling & testing	Syndromic management of STI^*^	IEC^**^ on STI/HIV	Clinical management of rape survivors
	%	%	%	%
Capital	15.6	32.9	51.2	27.3
Central Highland	32.1	53.4	56.6	51.
Eastern	14.3	40.4	44.3	31.8
Northeastern	43	36.3	60.3	18.8
Southeastern	50.4	40.3	81.8	52.9
Western	16.3	36.5	41.9	35
Northern	15.9	38.2	56.8	16.1
Southern	24.5	44.6	55.1	64.7

* STI, sexually transmitted infection.

** IEC, Information, education & communication.

Regarding the clinical management of rape survivors, the highest provision is observed in the Southern region at 64.7%, followed by the Southeastern region at 52.9%, while in the Northeastern and Northern regions, availability ranges from 16 to 18%.

**Figure 9:**
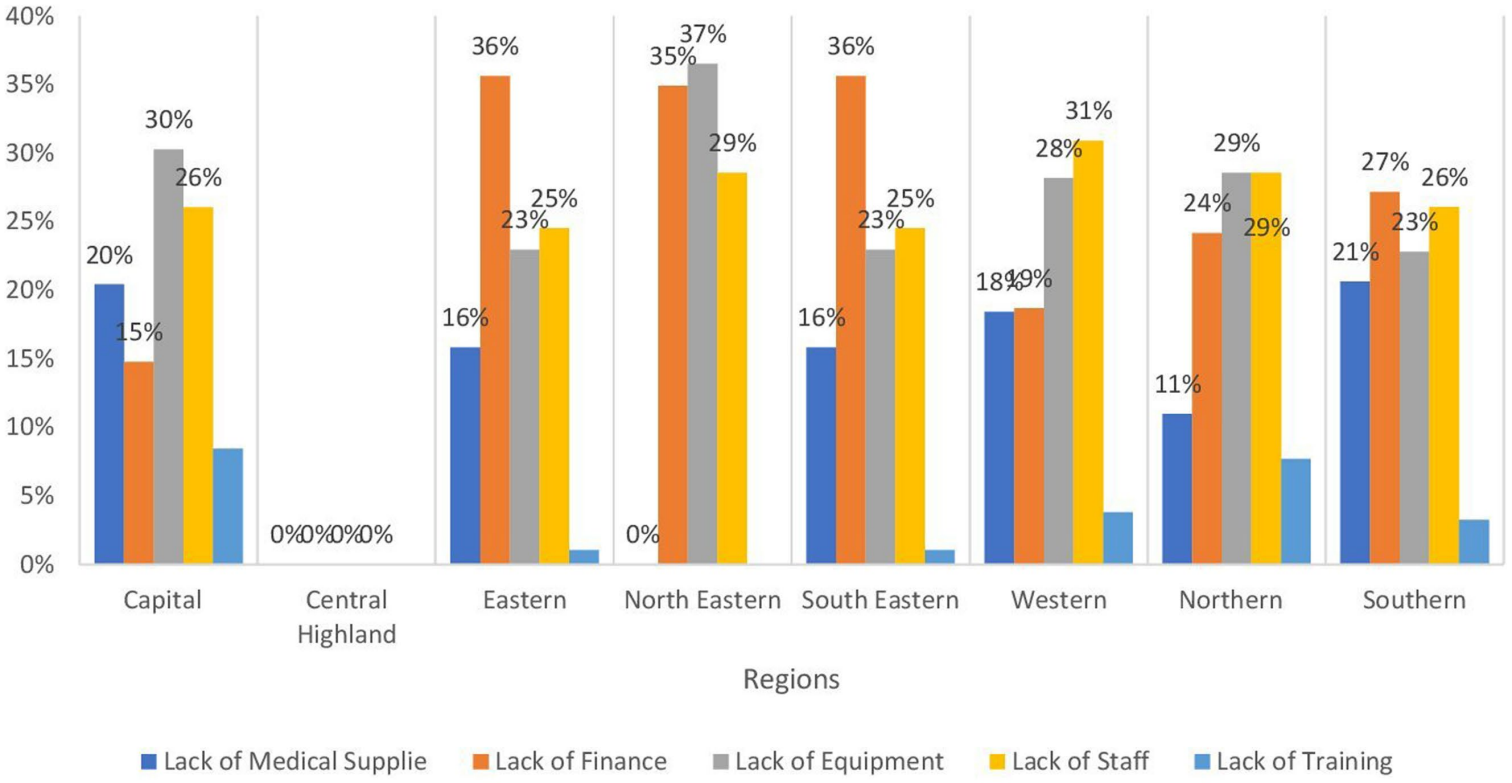
Bar chart demonstrating the main barriers to the availability of basic laboratory services by regions.

Table 8 presents the availability and accessibility of essential diagnostic services, which is a cornerstone of a functioning healthcare system, revealing generally low availability across regions, with percentages slightly exceeding 20% or less. Basic laboratory services display higher availability in the Capital region at 23%, with lower availability across the remaining regions ranging from 10 to 20%. The significantly limited availability of fundamental laboratory services is largely attributed to financial constraints, accounting for nearly 36% in both the Eastern and Southeastern regions. Moreover, in the Northeastern region, the lack of equipment also contributes significantly, representing 37% of the barriers to availability, as evidenced in Figure 9. In the Northeastern region, the availability of laboratory services at the secondary level is noted at 16.9%, with lower availability across the remaining regions ranging from 5.5% in the Southern and Central Highlands to 11% in the Capital region. Similarly, laboratory services at the tertiary level are available in the Southeastern region at 16%, with lower availability across the rest of the regions, reaching the lowest percentage in the Central Highlands at 0.8%.

**Table 8:** Percent distribution of the availability of diagnostic services for diseases across regions in Afghanistan (*n* = 4,760).

Region	Percent of the availability of Diagnostic services				
	Basic laboratory	Laboratory services at the secondary level	Laboratory services at the tertiary level	Basic X-ray services	Endoscopic services
	%	%	%	%	%
Capital	23.4	11.7	3.8	8.6	0.5
Central Highland	12.5	5.6	0.8	3.2	0
Eastern	20.1	8.1	4.4	3.2	0.2
Northeastern	20.8	16.9	7.8	3.7	0.4
Southeastern	19.9	6.3	16.1	3.6	0.2
Western	10.5	7.9	2.9	3.1	0.2
Northern	11.5	6.4	4	4.5	0.7
Southern	15.4	5.5	2.9	1.9	0.2

The availability of basic X-ray services is less than 10%, with the highest availability in the Capital region at 8.6% and ranging from 1.9% in the Southern region to 4.5% in the Northern region.

Furthermore, the availability of endoscopic services is less than 1% in all regions and is not available in the Central Highlands region.

Table 9 provides an analysis of the availability of fundamental amenities across various regions, such as water, electricity, sanitation, and waste management, which are essential for promoting public health, supporting a healthy population, and facilitating effective healthcare delivery. Water availability demonstrates high prevalence in most regions, ranging from 86.5% in the Southeastern region to the highest at 92.1% in the Northeastern region. The Western and Northern regions exhibit slightly lower availability at 68.7 and 65.9%, respectively. Similarly, power availability shows high prevalence, ranging from 86.3% in the Southern region to the highest at 94% in the Capital region, with slightly lower availability in the Western (77.4%) and Southeastern (79.1%) regions. As for cold chain availability, the Southern region demonstrates the highest availability at 79.4%, followed by the Southeastern and Northeastern regions at nearly 78%, while the Central Highland region has the lowest availability at 60.9%.

**Table 9:** Percent distribution of the availability of basic amenities across regions in Afghanistan (*n* = 4,760).

Regions	Basic amenities					
	Water availability	Power availability	Cold chain availability	Sanitation facilities availability	Waste segregation availability	Final disposal of infectious waste availability
	%	%	%	%	%	%
Capital	90.1	87.2	74.8	77.4	87.9	88.9
Central Highland	88.2	93.1	60.9	84.5	97.1	95.5
Eastern	88.1	88.6	73.9	82.1	87.6	85.9
Northeastern	92.1	94	77.4	89.2	88.8	88.6
Southeastern	86.5	86.3	78	83.9	86.9	88.3
Western	68.7	79.1	65.3	66.8	73.9	72.6
Northern	65.9	77.4	66.1	61.1	85.3	87.4
Southern	87	90.1	79.4	87.1	91.1	93.4

In terms of sanitation facilities, availability surpasses 70% across Afghanistan, with the highest observed in the Northeastern region at 89.2%, while the Northern and Western regions exhibit lower availability at 61.1 and 66.8%, respectively. Waste segregation and final disposal of infectious waste are both available at levels exceeding 70% in all regions, with the highest prevalence in the Central Highland region at 97.1 and 95.5% respectively, and the lowest in the Western region at nearly 73%.

## 3 Discussion

Our findings reveal a complex picture of healthcare provision across various regions, with a significant reliance on sub-health centers and basic health centers, coupled with the crucial role of mobile health teams in reaching geographically dispersed populations. However, there are notable challenges in the availability of critical services like emergency care, intensive care, and blood bank services. Additionally, the significant impact of financial constraints and equipment shortages on the functionality of healthcare facilities exists particularly in the Northeastern and Western regions.

The proportion of male health care workers (HCWs) was higher than females (1.5 times). However, the distribution in different fields varied where the majority of pharmacists and technicians were males while almost all midwives were females. The Ministry of Public Health in the country alongside international organizations reintroduced the training and capacity building of female midwives aiming at increasing the proportion of female HCWs (8). As it is still culturally unacceptable in many areas for a female to be examined by male HCW (2).

Health facilities providing services vary in the country, where around 50% of the services are provided by either sub-health centers or basic health centers. This number is consistent with the percentage of permanent or fixed health structures which represents around 54%.

However, due to the geography of the country having many mountainous areas (10) with individuals distributed over multiple small terrains and the high potential of mobilization (10, 11). the presence of mobile health centers is also mandatory which represents 17% which is also close to the percentage of mobile clinics (16%). With over three-quarters of the population living in rural areas, the role of mobile clinics is critical to reach these individuals (12).

More than half of the health facilities are funded by the World Bank through the Health Emergency Response program. This figure is extremely important as it subjects the country to major consequences in case of any partial or complete withdrawal of the funds due to any global crisis.

Regarding the functionality of healthcare facilities, the percentages of fully functioning and ones with insignificant damage varied across the country, yet the majority were functioning properly with minimal damage. The Western region was the least one having facilities with insignificant damage (65%) and the second region with the least fully functioning facility after the Capital region. Even though the capital region has the least fully functioning facilities, it is ranked second in facilities with insignificant damage. Furthermore, there were low percentages of non-functioning facilities which are either not present or below 1% with the only exception in the capital region with eight non-functioning facilities. In addition, facilities with major or fully damaged accounted for less than 0.5% across different regions. The northeastern region has the highest percentage of partially functioning facilities. The main reasons behind the low figures in the latter region are insufficient financing and inadequate equipment. The percentages of mobile clinics ranged from seven to 16% across the country. This variation could be due to the nature of each region making it easier or harder to reach certain provinces.

In terms of the supply of clinical services, general clinical services provision in the country showed a wide variation across regions and types of services. Outpatient services displayed relatively higher figures in most regions whereas the percentages of emergency or inpatient services were skewed to the other side. The central highland was the most affected region with lower coverage in the majority of services. Regarding outpatient services for primary care, it was over 64% in the country with the northeastern region having the highest coverage whereas the capital and central highland had the lowest one. The predominant reasons for the low coverage in the Capital and Central highlands are inadequate medical supplies and insufficient funds. The percentages showed a minor decrease from primary to secondary care services at the outpatient clinics except for the Western region which showed a minimal increase. It was noted that the only wide gap was in the central highland indicating a lack of specialists in the region. However, the latter region showed a much higher percentage in the home visits compared to the other regions which could compensate for the lower rates of outpatient services. Moreover, it is important to indicate that even if some figures are inappropriate, there has been a documented increase in the uptake of services in both outpatient and inpatient during the first half of 2021 and 2022 (13).

In areas with a high potential for conflicts and unstable conditions, the presence of emergency and intensive care services is of great value (10). It is estimated that around one-third of the country on average is covered by intensive care services being highest in the southeastern region (44%) whereas it was lowest in the eastern region (9%). This indicated that even though the recognition of dangerous symptoms and signs was over 50 in any region, yet the low coverage of intensive and emergency services rendered any intervention questionable even in the presence of a functioning referral system which may have a role in the outcome of any service delivery. The low percentages of blood bank services (less than 6%) in any region could explain the reason of the low coverage and effectiveness of intensive care services, surgeries, and invasive interventions. This could also explain the low coverage for WHO emergency interventions. Consequently, there is a need to adopt different approaches to deal with conflicts and unstable conditions to improve emergency and intensive care services (13, 14).

Under five children is one of the important vulnerable groups and providing them with the required services is crucial and proven to be cost-effective. One important service is basic vaccinations. In the present research, wide variations in the EPI were noted where the Western region had the lowest coverage percentage (46%) which is attributed mainly to the lack of assigned staff, medical supplies, and equipment. The northern region also showed low figures at 48% where the main obstacles were lack of staff members and financial support. On the other side, the southern region showed the highest coverage (79%). These percentages tend to be close to the coverage rates for the community mobilization for EPI except for the central highland which showed around 13% increase. These numbers present a major threat and alarming signal to the health of this vulnerable group as from 11 to 53% were not covered by basic vaccination where previous research indicated that only 18% of children are fully immunized (15). This exposes them to major morbidities and mortalities from these communicable diseases even though the cold chain availability across the country showed much higher coverage percentages (61% in central highland to 79% in Southern regions). Regarding other under-five services, there are discrepancies according to the type of service where figures showed higher coverage for screening, routine follow-up, IMCI, and management of moderate and severe malnutrition at the outpatient clinics with higher levels of information, education, and communication for this group where the percentages exceeded 70% in most categories and regions. Factors affecting coverage varied across regions. For example, the main obstacle to providing IMCI was the inadequacy of medical supplies in the Capital region while it was the deficiency of training in the Eastern one. However, the coverage rates showed marked low coverage for management of severe diseases alongside inpatient management of severe malnutrition problems which were lower than 50% for the former and 20% for the latter. This again sheds light on the difficulties in providing services related to emergency and inpatient departments mentioned before. Moreover, food insecurities could be a major role player in malnutrition and mortality from undernutrition especially taking into consideration that it is estimated that around 20 million citizens are in need of food assistance (16). In addition, the United Children’s Fund (UNICEF) stated that there are around 10 million children in Afghanistan in need of humanitarian assistance (17).

This study outlines prenatal, natal, and postnatal services provided in Afghanistan for women who are childbearing period All regions showed antenatal coverage of over 80% with the Capital region being the only exception (72%), where the main reason being the scarcity of medical supplies in this region. Delivery by skilled birth attendants showed similar figures as antenatal coverage. The exceptions were noted in the Eastern region (16% decrease) and Western region (9% decrease). Inaccessible or remote locations though reported few increases in the count of trained birth attendants in other research (18). The Eastern region also showed a much decrease in percentage regarding clean home delivery being the least one (51%). Regions varied in the percentages of clean home deliveries especially that this was also the case for basic emergency obstetric care where it was noticed that almost 50% did not have either clean home delivery or basic obstetric care in the Capital and Eastern regions, yet these figures showed much improvement in other regions. The low coverage percentages of emergency obstetric care in the Capital and eastern regions are caused mainly by insufficiently trained staff followed by deficiency of medical supplies followed by the absence of financial support. A marked decrease in emergency obstetric care was found when a higher level of care is required; a comprehensive one which agreed with the previously mentioned figures of secondary level of care for the entire population. Considering that the main causes of maternal mortality are preventable such as bleeding and obstructed labor (19) the presence of secondary or specialized care is important to decrease maternal mortality rates as well as increasing the availability of blood banks where the coverage percentages for both showed low figures.

Referring to antenatal care, postpartum care, and family planning, which showed higher coverage exceeding 70% in any region, with the Capital region being the least covered, it is important to mention that utilization of family planning services has improved compared to previous years (20, 21).

.Control of communicable diseases is of great importance, especially in developing countries like Afghanistan. Vector control as a primary preventive measure for arthropod-borne infection was lowest in Northern and Western regions (7 and 10% respectively) while it was highest (over 43%) in Central and Eastern regions. The latter region’s relatively higher percentage could be explained by the high burden of arthropod-borne hemorrhagic fever such as dengue in provinces like Nangarhar (22). Syndromic surveillance averaged around 30% in most regions, being highest in Central Highland (43%) and lowest in Eastern and Capital regions (14 and 21% respectively). Secondary prevention at the PHC level was recorded for malaria and tuberculosis. The former was lowest in Western and Northern regions (below 60%) while it was highest in Central, Northeastern, and Southeastern (above 78%). As for tuberculosis, almost two-thirds of the population was not covered by services at the PHC level, and this number increased for multi-drug resistance strains with exceptions in southeastern, western, and southern regions. As the disease is difficult to be diagnosed, the lack of health promotion activities in the country (23) increases the disease burden. This is very important and impending outbreaks are probable due to the low coverage for vector control. Around half of the population had proper management for complicated communicable diseases at most and 23% at least in central and northern regions. This could be explained by the low availability of isolation facilities for communicable diseases which were below 20% in all regions except for Capital one.

The growing burden of non-communicable diseases over the past years requires efforts, especially with the increase in life expectancy. However, in the present current research, less than 50% were provided with NCD clinics which was lowest in Western and Northern regions (less than 23%). Moreover, diabetes mellitus coverage was as low as less than one quarter except for capital and central highland which was below 30%. These percentages increased for COPD and asthmatic patients while it was highest for hypertension. A report published in the country showed that NCDs burden is growing and the majority of the DALYs is caused by cardiovascular diseases, COPD, neoplasms, diabetes, and digestive diseases (24). In addition, the former four diseases have a major impact on mortality in Afghanistan (25). Despite these figures, non-communicable diseases have not gained much of the attention from either government or donors (26).

Non-communicable diseases are known to have several complications making tertiary prevention of great value yet, the coverage for inpatient rehabilitation and different physiotherapy services was very low being less than 20% for the former and 10% for the latter in most provinces. Regarding management of mental disorders, the coverage ranged from 21 to 52% with around one-third of the population having access to psychological first aid. This is of extreme importance as a recent survey revealed that over half of the participants showed little to extreme concerns after the US withdrawal from the country (27). Furthermore, the country shows high rates of mental problems and disorders compared to the outside world (28). The inpatient mental disorders services coverage was low as most of the inpatient services. The reasons behind the low availability of mental services in the central highland and Northern regions are the absence of staff in the former which was the same reason along with the absence of supplies, financial, and training in the latter.

Sexually transmitted infections (STIs) syndromic management coverage ranged from one-third of the population in the Capital region to around half of the population in the central highland. However, HIV testing and counseling was below 25% in five regions. Even though information, education, and communication for the disease were much higher reaching up to 82% in the Southeastern region. This could indicate a gap between demand or needs from one side and supply from the other side. It was also noted that there were variations within different regions regarding the management of rape survivors indicating the possibility of cultural or stigma barriers.

On the other hand, confirmation of any disease or health problems relies mainly on investigations either laboratory or radiological. Relying upon clinical examination could result in an inaccurate estimation of the prevalence or incidence of any health condition. In the current research, laboratory investigations coverage showed low figures where less than one-quarter of the population had access to basic laboratory services in the Capital region which further decreased to be lowest in the Western region (11%). Financial constraints were the major obstacle in the Eastern and Southeastern regions whereas, in the Northeastern region, the lack of medical equipment was the main reason.

These figures showed a much decline (below 17%) for the secondary level with most of the regions having less than 10% coverage. The coverage decreases much more for the tertiary level. In the same context, basic X-ray services coverage was below 5% in all regions except for the capital one which showed minimal increase. It was also noted that any endoscopic services were below 1% in the country.

Environmental sanitation has a major impact on the prevention and control of multiple morbidities. That’s why ensuring the sustainability of the provision of public services is fundamental for improving the living conditions in the most needed areas. This commitment includes fields like water sanitation, electricity supply, and other basic needs (29). Water availability across the country surpassed 85% with exceptions in Western and Northern regions (69 and 66% respectively). The same was found regarding sanitation facilities. The entire region though showed higher percentages regarding waste segregation and infectious waste disposal. Power availability exceeded 77% in all regions.

## 4 Conclusion

The present study has endeavored, through the utilization of The Health Resources and Services Availability Monitoring System (HERAMS), to assess the availability and allocation of healthcare services and resources across various regions in Afghanistan. While most facilities were fully operational, the Northeastern and Western regions exhibited higher rates of partial damage and dysfunction, primarily due to financial constraints and equipment shortages. Notably, critical services like emergency care, intensive care, and blood bank services displayed low coverage nationwide, highlighting a need for focused interventions. The availability of essential child health, maternal health, and communicable disease services varied widely across regions, with significant gaps in the Western region. This study underscores the need for targeted resource allocation, particularly to underserved regions, to improve access to essential healthcare services and achieve equitable health outcomes in Afghanistan.
